# Hit the mark with diffusion-weighted imaging: metastases of rhabdomyosarcoma to the extraocular eye muscles

**DOI:** 10.1186/1471-2431-14-57

**Published:** 2014-02-27

**Authors:** Nicole Hassold, Monika Warmuth-Metz, Beate Winkler, Michael C Kreissl, Karen Ernestus, Meinrad Beer, Henning Neubauer

**Affiliations:** 1 Department of Paediatric Radiology, Institute of Diagnostic and Interventional Radiology, Josef-Schneider-Straße 2, Wuerzburg 97080, Germany; 2 Department of Neuroradiology, Institute of Diagnostic and Interventional Radiology, Josef-Schneider-Straße 11, Wuerzburg 97080, Germany; 3 Department of Paediatrics, Josef-Schneider-Straße 2, Wuerzburg 97080, Germany; 4 Department of Nuclear Medicine, Oberduerrbacher Straße 6, Wuerzburg 97080, Germany; 5 Institute of Pathology, Josef-Schneider-Straße 2, Wuerzburg 97080, Germany

**Keywords:** Rhabdomyosarcoma, Metastases, Extraocular eye muscles, DWI, PET/CT

## Abstract

**Background:**

Rhabdomyosarcoma is the most frequent malignant intraorbital tumour in paediatric patients. Differentiation of tumour recurrence or metastases from post-therapeutic signal alteration can be challenging, using standard MR imaging techniques. Diffusion-weighted MRI (DWI) is increasingly considered a helpful supplementary imaging tool for differentiation of orbital masses.

**Case presentation:**

We report on a 15-year-old female adolescent of Caucasian ethnicity who developed isolated bilateral thickening of extraocular eye muscles about two years after successful multimodal treatment of orbital alveolar rhabdomyosarcoma. Intramuscular restricted diffusion was the first diagnostic indicator suggestive of metastatic disease to the eye muscles. DWI subsequently showed signal changes consistent with tumour progression, complete remission under chemoradiotherapy and tumour recurrence.

**Conclusions:**

Restricted diffusivity is a strong early indicator of malignancy in orbital tumours. DWI can be the key to correct diagnosis in unusual tumour manifestations and can provide additional diagnostic information beyond standard MRI and PET/CT. Diffusion-weighted MRI is useful for monitoring therapy response and for detecting tumour recurrence.

## Background

Rhabdomyosarcoma is the most frequent malignant intraorbital soft tissue tumour in paediatric patients [1]. Primary orbital manifestation is considered to be associated with a relatively good prognosis, while extraorbital extension, the alveolar subtype and orbital relapse are associated with an unfavourable outcome. Classical applications of diffusion-weighted MRI (DWI) include imaging of acute ischaemic insult, cerebral abscess and, more recently, differentiation of tumour entities. Recent studies suggest that DWI holds potential for better differentiation between benign and malignant intraorbital masses [2].

## Case presentation

**Figure 1:**
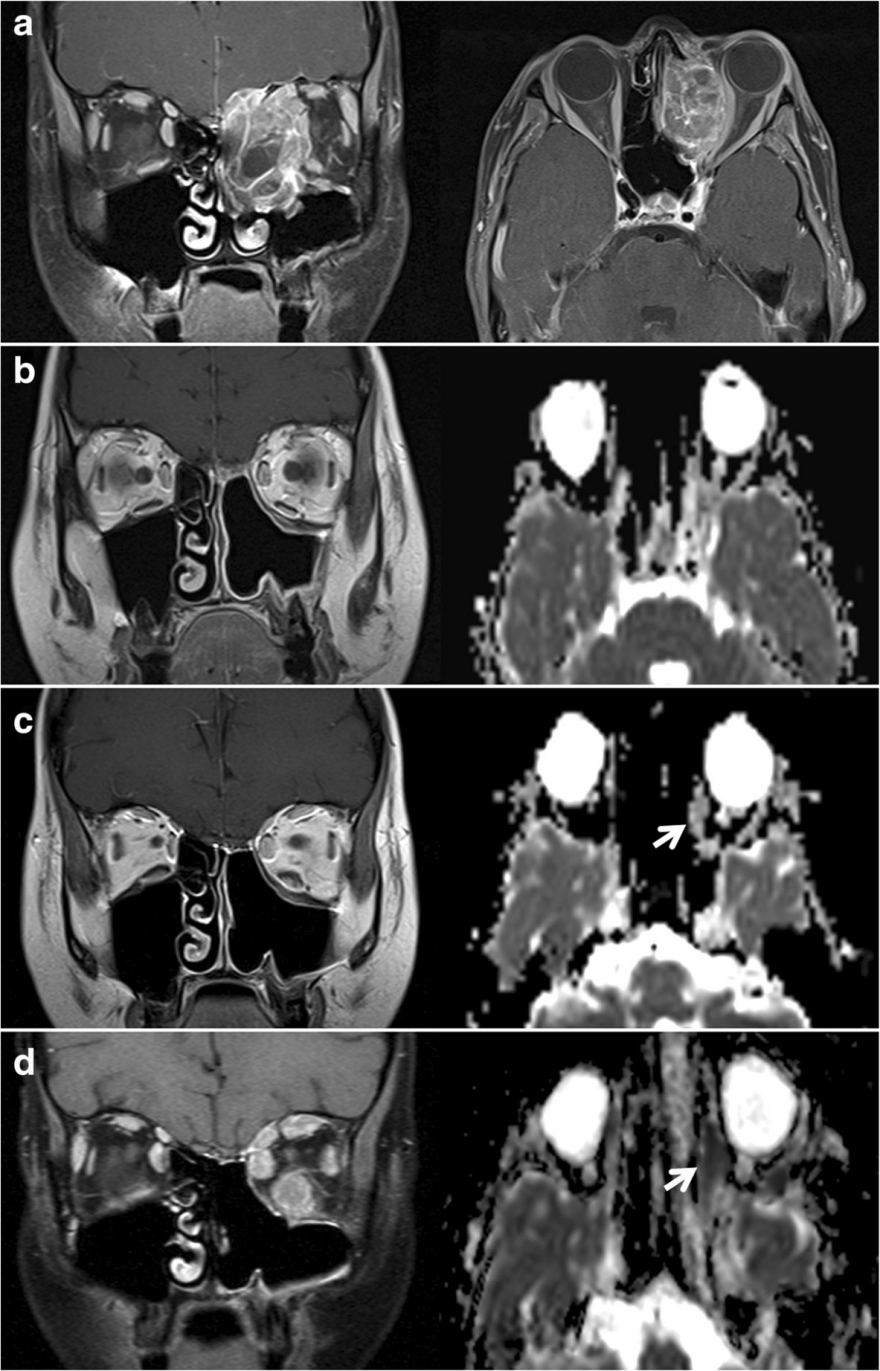
**Longitudinal MRI studies of the orbitae. (a)** Initial diagnosis of the left-orbital rhabdomyosarcoma with infiltration of the ethmoidal air cells and the left maxillary sinus (coronal and transversal contrast-enhanced fat-saturated T1w). **(b)** Normal post-therapeutic follow-up study. **(c)** At onset of double vision, discrete thickening of the left medial and inferior rectus muscle in the coronal T1w and of the left lateral rectus muscle in the ADC-map (arrow) became apparent, yet without pathological decrease in diffusivity (ADC = 1.25 x10^−3^ mm^2^/s). **(d)** Follow-up MRI showed progressive signal alterations in all left extraocular eye muscles and initial changes in the right medial rectus muscle. Both left and right medial rectus muscle showed significantly restricted diffusion (arrow, ADC = 0.62 x10^−3^ mm^2^/s). Figures 1b,c,d show coronal contrast-enhanced T1w and ADC-maps calculated from transversal DWI with b-values of 0 and 1000 s/mm^2^. The examinations were performed at 1.5 Tesla (1a and 1d) and at 3 Tesla (1b and 1c).

In April 2010, an otherwise healthy 15-year-old girl first presented with acute left-sided protrusio bulbi. MRI revealed an extraconal mass in the left superonasal orbital quadrant with infiltration of the paranasal sinuses and the skull base (Figure 1a). Endonasal biopsy confirmed alveolar rhabdomyosarcoma with PAX3-FKHR-translocation. PET/CT did not show evidence of metastatic spread, and the tumour was staged as T2 N0 M0. Chemotherapy, radiation treatment of the primary tumour and complete tumour resection followed. Five months after completion of treatment, follow-up imaging with PET/CT and MRI detected a solitary bone metastasis in the right femoral neck and two intramammary soft tissue metastases. Tumorous bone marrow infiltration of less than 1% was diagnosed in bone marrow specimen. Multimodal treatment, including chemotherapy, surgical resection of the metastases, local radiation and haploid stem cell therapy achieved complete response.

**Figure 2:**
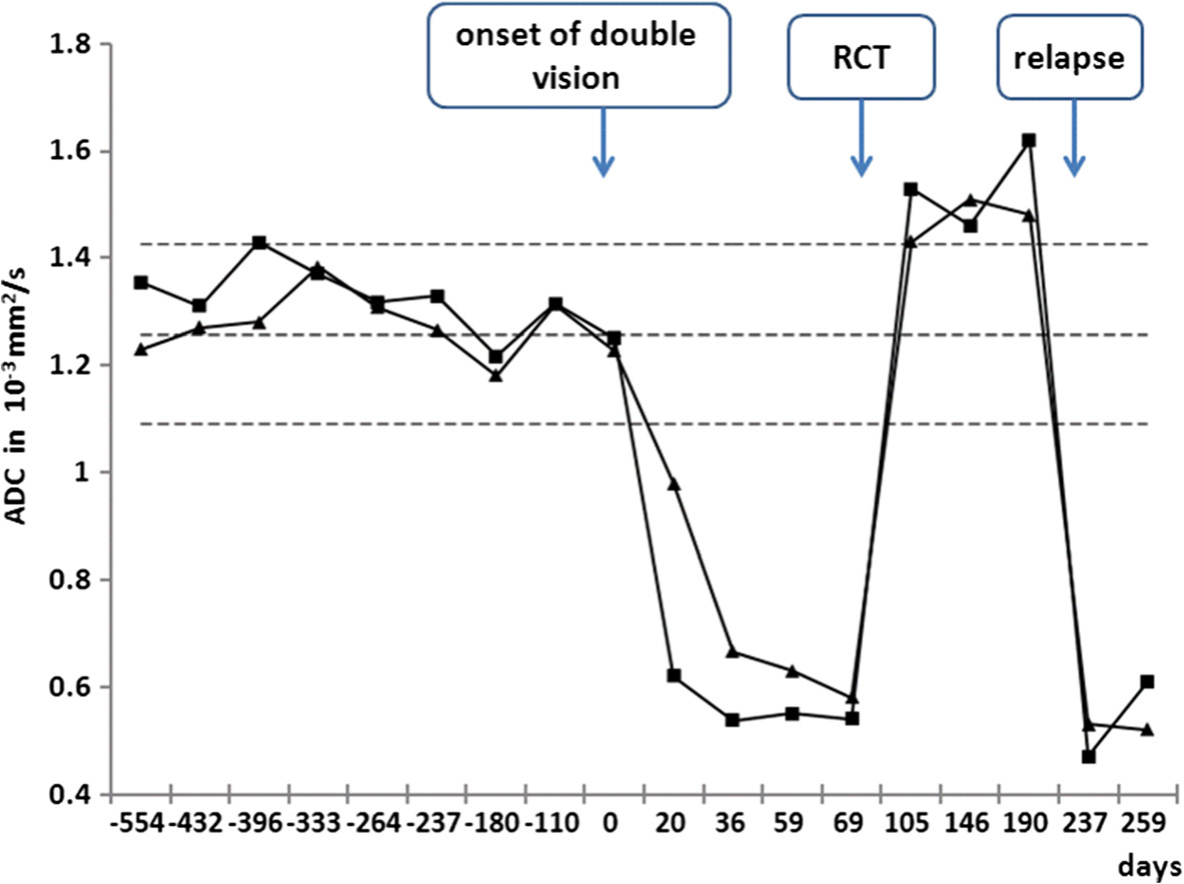
**Time course of ADC values in the extraocular eye muscles.** Mean ADC of left (■) and right (▲) medial rectus muscle before and after onset of current symptoms, under chemoradiotherapy and with tumour relapse. The dashed lines represent data (mean ADC values ± 2 SD) from a reference cohort of 20 patients without orbital pathology.

**Figure 3:**
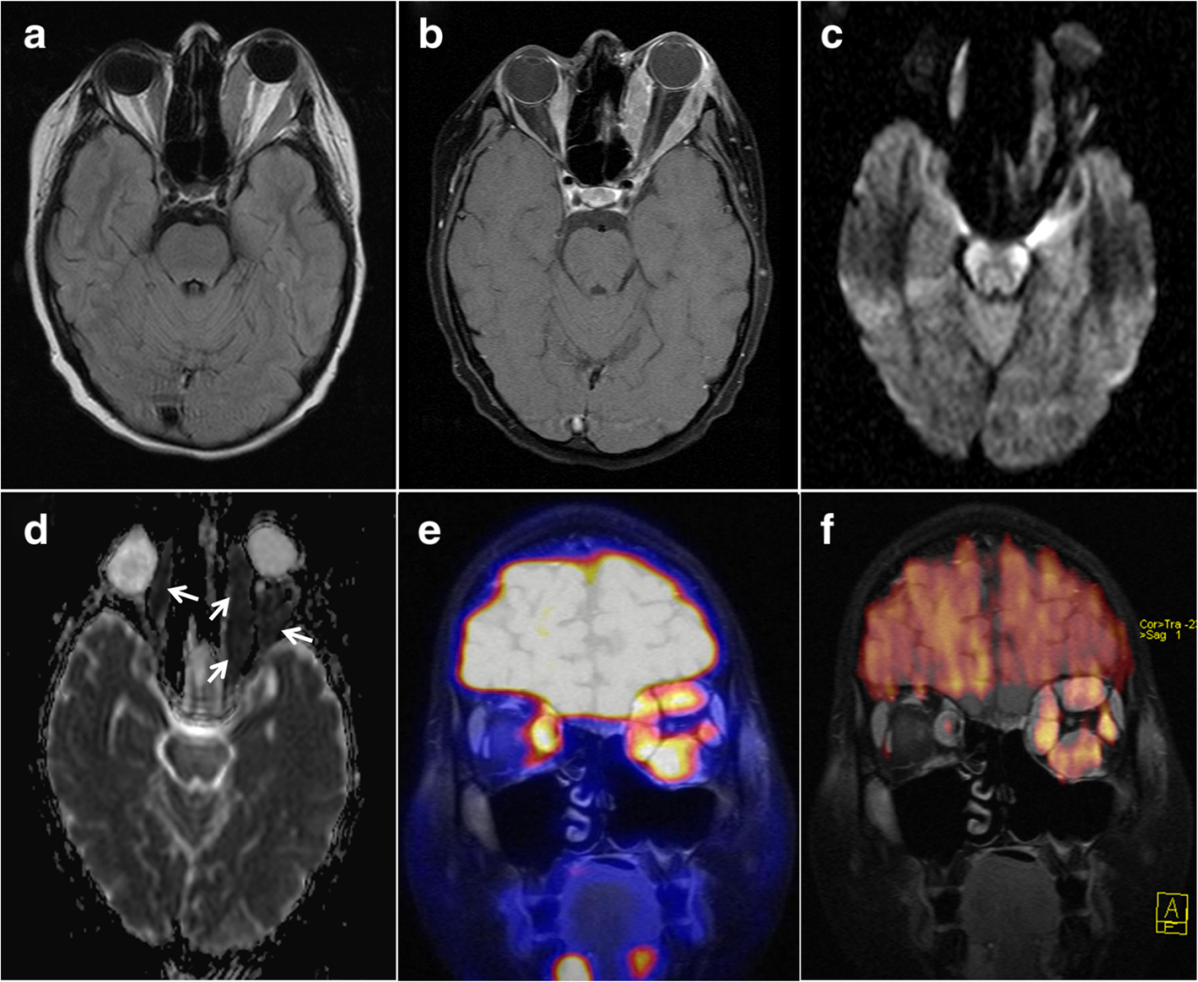
**Rhabdomyosarcoma metastases to the extraocular eye muscles.** Excessive bilateral thickening of extraocular eye muscles and left-sided exophthalmos two months after onset of symptoms. **(a)** pre-contrast T1w, **(b)** contrast-enhanced fat-saturated T1w, **(c)** DWI b = 1000 s/mm^2^ and **(d)** ADC map indicating markedly restricted diffusivity in the extraocular eye muscles (arrows), fusion of MRI (coronal contrast-enhanced fat-saturated T1w) with FDG-PET **(e)** and with colour-encoded DWI b = 1000 s/mm^2^**(f)**.

**Figure 4:**
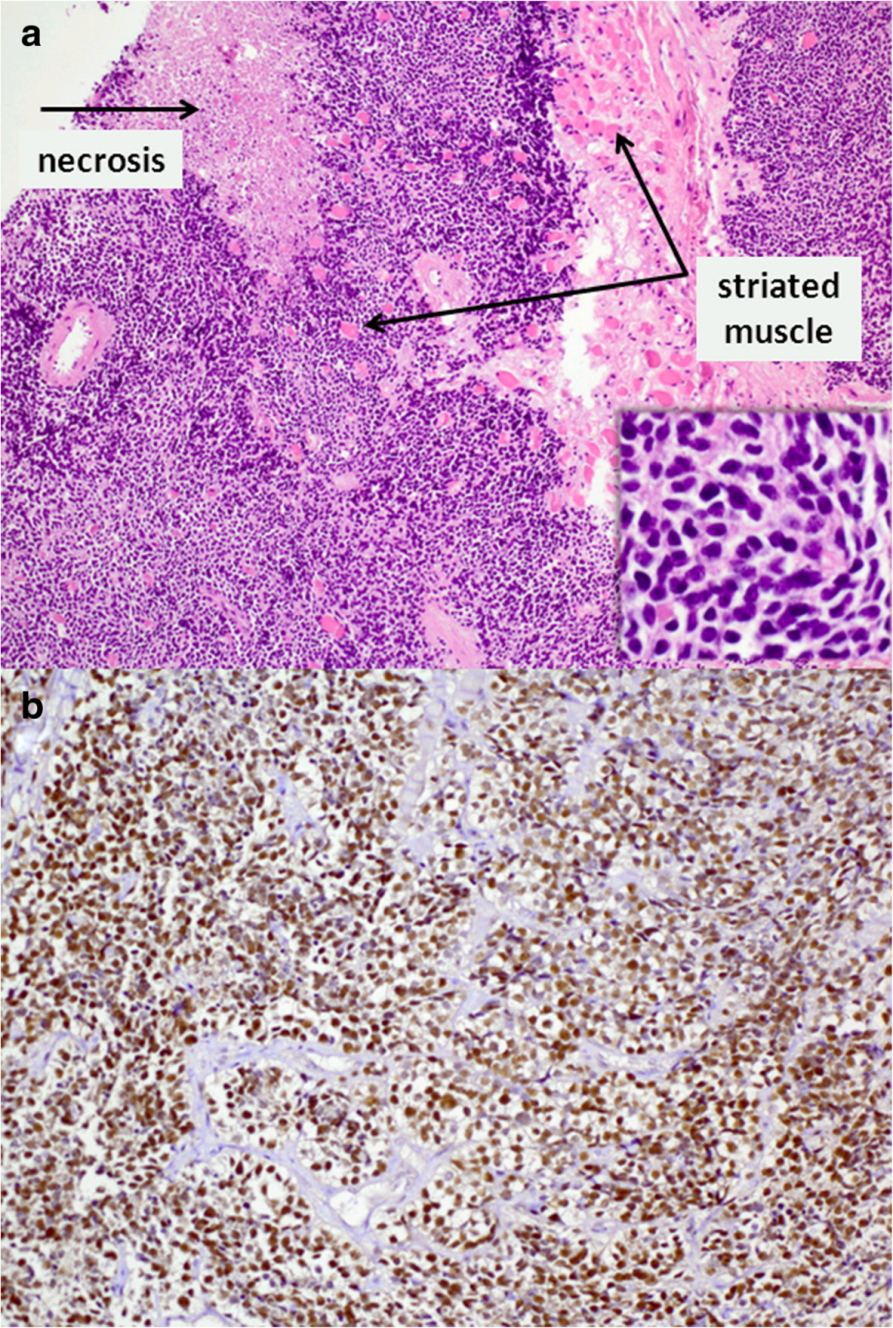
**Histopathological specimen from left inferior rectus muscle biopsy. (a)** Haematoxylin-eosin staining (× 100, detail enlargement × 400) showing infiltration by a small-blue-round-celled tumour, consistent with a metastasis of the known rhabdomyosarcoma **(b)** Immunohistochemical staining of tumour cells showing expression of myogenin (× 200).

In early 2012, three months post-therapy, the patient complained about newly occurred double vision. In comparison to numerous preceding MRI studies, a new, though initially very discrete, thickening of the left medial rectus and inferior rectus muscle was observed in the absence of other intraorbital signal alterations (Figure 1b, c). As clinical symptoms aggravated over the ensuing three weeks, we noted progressive bulging of all extraocular left extraocular eye muscles and thickening of the right medial rectus muscle. MRI signal alterations included homogenous T2w signal elevation, increased uptake of contrast media and increasingly restricted diffusivity on follow-up MRI examinations (1.5 Tesla Magnetom Symphony and 3 Tesla Trio, Siemens Medical, Germany, diffusion-weighted single-shot echoplanar SS-EPI imaging with b-values of 0 and 1000 s/mm^2^; at 1.5 Tesla TR 4600 ms, TE 137 ms, no parallel imaging, at 3 Tesla TR 3300 ms, TE 90 ms, parallel imaging iPAT = 2). Restricted diffusion was indicated by progressively high intralesional signal on DWI at b = 1000 s/mm^2^ and a corresponding drop in ADC values (apparent diffusion coefficient, unit 10^−3^ mm^2^/s) from 1.25 (long-standing baseline) to 0.6 × 10^−3^ mm^2^/s in the left medial rectus muscle within three weeks. A similar time course of ADC in correlation to progressive muscular thickening was observed in all affected eye muscles (Figure 1d, Figure 2 day 20 to day 36). Whole-body FDG-PET/CT visualised intralesional tracer uptake exceeding physiological muscular signal in the absence of suspicious extraorbital lesions. Open biopsy of the left inferior rectus muscle was performed seven weeks after onset of double vision. Histological evaluation initially showed fibrosis and interstitial oedema without signs of tumour infiltration. Further serial sectioning and additional immunohistochemical staining revealed a small number of scattered atypical cells suggestive of rhabdomyosarcoma. The patient suffered further clinical deterioration with massive exophthalmos and impaired vision of the left eye, accompanied by further progression on MRI with persistently low ADC values (Figure 3, Figure 2 day 59). Eleven weeks after the onset of orbital symptoms, operative transnasal decompression of the left orbita became necessary in order to prevent loss of vision. Intraoperative biopsy showed extensive tumour infiltration of striated muscle and surrounding fatty tissue by small round blue cells with alveolar growth pattern, expression of myogenin, focal positivity for desmin and weak expression of MyoD1 (Figure 4). Oral chemotherapy and radiation of both orbital cavities resulted in complete tumour remission and elevated muscular ADC above baseline level up to oedema-equivalent values (1.4 ~ 1.6 × 10^−3^ mm^2^/s) (Figure 2 day 105). However, after another fifteen months and two more local recurrences treated with palliative chemoradiotherapy, the patient eventually suffered diffuse systemic metastatic spread and deceased in August 2013. The first intramuscular tumour relapse after three months of complete remission (Figure 2, day 237), the subsequent episodes of local recurrence and the eventual systemic spread (ADC values not shown in Figure 2) were again all characterised by high DWI signal and very low intralesional ADC ranging between 0.5 and 0.9 × 10^−3^ mm^2^/s.

## Conclusions

Rhabdomyosarcoma metastases confined to the extraocular eye muscles constitute a rare clinical entity [3]. In our case, the peculiar intramuscular signal alterations observed in the absence of any other orbital or systemic abnormalities posed a diagnostic problem for standard imaging techniques. As these signal alterations were most pronounced in the medial rectus muscles bilaterally, that is, closest to the primary radiation field, clinical differential diagnosis first considered a sequel of preceding radiotherapy. Furthermore, endocrine orbitopathy and immunoreactions secondary to stem cell transplantation were discussed. SUV (standardised uptake value) quantification on FDG-PET/CT was not found helpful to further clarify the aetiology of muscular swelling, as normal eyes muscles show high physiological FDG uptake and as inflammation could not be ruled out as a cause of increased tracer update in the affected muscles. While findings on standard MRI sequences were initially considered as non-specific, the early finding of restricted diffusion in the affected eye muscles on DWI clearly argued against an inflammatory aetiology (Figure 2 day 20 and 36). According to our own experience with extracranial DWI, inflammatory and oedematous soft tissue lesions present with a relatively stereotypic increase in ADC ranging from 1.4 to 2.6 [4]. ADC values < 1.0 measured in osseous or soft tissue lesions are characteristic of malignant tumours with high cellularity, abscess und some classical findings, such as epidermoid tumours [5]. In the presented case, increasingly high intramuscular signal on DWI at high b-values with corresponding low ADC values was the earliest diagnostic indicator of malignancy.

Some limitations of our study deserve consideration. Firstly, MR imaging at the time of primary diagnosis did not include DWI, so information on diffusivity in the primary tumour is not available. Secondly, some rhabdomyosarcoma may exhibit higher ADC [2,6,7]. While the characteristic low ADC values noted in this study underline a high specificity for malignant tumour, sensitivity for rhabdomyosarcoma in general may be limited to some extent. Thirdly, the series of MRI scans in this case was performed in turns on two MRI scanners. We are aware that this may have caused some degree of variation in longitudinal ADC values. Still, we used scanner hardware of the same manufacturer and similar scan protocols on both platforms. In our experience, other sources of error, such as manual placement of ROI for ADC quantification, account for a much higher degree of variation in ADC data.

In summary, diffusion-weighted MRI is increasingly regarded as a helpful tool in the diagnostic work-up of orbital tumours. Promising current data from the head-neck-region show a diagnostic benefit with improved differentiation of malignant and benign neoplasm [6,8]. In a recent study on orbital tumours conducted with DWI at 3 Tesla, sensitivity, specificity and diagnostic accuracy for differentiation of malignant from benign disease were reported as 95%, 91% and 93% [9]. The presented case demonstrates that diffusion-weighted MRI can provide crucial information for establishing the correct diagnosis at an early stage and may prove particularly useful in patients with unusual findings in uncommon localisations. In general, “bright spots” on diffusion-weighted images with corresponding low signal on ADC maps warrant close attention and may likely be the key to correct diagnosis and timely treatment.

## Consent

Written informed consent was obtained from the legal guardians of the patient for publication of this Case report and any accompanying images. A copy of the written consent is available for review by the Editor of this journal.

## Abbreviations

ADC: Apparent diffusion coefficient; DWI: Diffusion-weighted magnetic resonance imaging; FDG: Fluordeoxyglucose; MRI: Magnetic resonance imaging; PET/CT: Positron emission tomography/computed tomography; SUV: Standardized uptake value.

## Competing interests

All authors declare that they have no competing interests.

## Authors’ contributions

In detail: MB and HN conceived and outlined the case presentation. NH, MWM, MB and HN collected and analysed the MRI data. MCK collected and analysed the PET/CT data. BW was responsible for compiling clinical background information. HN and MCK performed image analysis and prepared imaging data and figures. KE contributed histopathological data and microscopy images. NH and HN drafted the manuscript. All authors revised the draft and agreed on the final version.

## Pre-publication history

The pre-publication history for this paper can be accessed here:

http://www.biomedcentral.com/1471-2431/14/57/prepub
